# Losing the desire: selection can promote obligate asexuality

**DOI:** 10.1186/1741-7007-8-101

**Published:** 2010-07-28

**Authors:** Kayla C King, Gregory DD Hurst

**Affiliations:** 1 Department of Biology, Indiana University, Bloomington, IN 47405, USA; 2 Institute of Integrative Biology, University of Liverpool, Liverpool L69 7ZB, UK

## Abstract

Whilst parthenogenesis has evolved multiple times from sexual invertebrate and vertebrate lineages, the drivers and consequences of the sex-asex transition remain mostly uncertain. A model by Stouthamer *et al. * recently published in *BMC Evolutionary Biology *shows a pathway by which obligate asexuality could be selected for following endosymbiont infection.

See research article http://www.biomedcentral.com/1471-2148/10/229

## 

Although sexual reproduction is costly, it is extremely widespread and is the dominant and ancestral mode of reproduction of eukaryotic organisms [1]. It is thus clear that sex must confer great benefits, and parthenogenesis (a type of asexual reproduction) is disadvantageous [2]. Despite their low evolutionary potential, parthenogenetic lineages do 'spin off' from sexual ancestors in a broad range of animals [3]. Several vertebrate and invertebrate species are composed of both sexual and asexual lineages. Stouthamer *et al. *[4] have now published a model of how obligate asexuality could evolve in parasitoid wasps with the help of a symbiont.

## Routes to parthenogenesis

There are several evolutionary routes to parthenogenesis in animals [3] (Box 1). One of the more remarkable recent experiments on the origin of asexuality demonstrated that certain haplodiploid taxa could be 'cured' of asexuality when treated with antibiotics [5]. Haplodiploid taxa, which include many social insects such as termites and wasps, produce males from unfertilized eggs and females from fertilized eggs. This demonstration [5] was explained by the fact that parthenogenesis can arise from manipulation by endosymbionts.

This system is now one in which the forces driving the establishment of asexual lineages are well known. Bacterial symbionts (such as *Wolbachia*, *Cardinium *and *Rickettsia*) can be transmitted only through the cytoplasm of eggs, from a female to her offspring. Males are essentially 'dead ends', and symbiont fitness is thus maximized by converting male progeny to female development. In arrhenotokous haplodiploid taxa (such as the parasitoid wasps studied by Stouthamer *et al. *[4,5]), this can be achieved by the parasite taking haploid unfertilized eggs that would develop into males and diploidizing their genome, such that they develop as females. At the start of this process, symbiont-infected females do mate with males, and fertilized eggs resulting from these matings develop into normal, sexually produced daughters. It is only the unfertilized haploid eggs that are subject to genome doubling and become asexually produced daughters.

### Box 1. Modes of parthenogenesis in animals

Organisms can possess lineages composed of facultative parthenogens (tychoparthenogens) or obligate parthenogens. In the case of the former, eggs infrequently and spontaneously develop without fertilisation, whilst in the latter 'cloning' is the only mode of reproduction. The forms of parthenogenesis include:

**Apomictic parthenogenesis **(apomixis) - meiosis is suppressed and unfertilized eggs develop into offspring by mitotic cell division. Apomixis occurs in rotifers and waterfleas.

**Automictic parthenogenesis **(automixis) - meiosis occurs, but diploidy is restored by duplication or fusion of gametes from the mother. Automixis is found in some stick insects and weevils.

**Gynogensis **- sperm from males of a related species activate egg development, but the sperm nuclei do not fuse with egg nuclei. Only maternal genes are expressed in the offspring.

**Hybridogenesis **- the genome is transmitted both sexually and clonally. Sperm and egg nuclei fuse and genes from the father are expressed in the offspring. When female offspring produce their own eggs, only the maternal genome is transmitted to the next generation; the father's genes are discarded. Hybridogenesis can occur in organisms that are hybrids of two species.

If unfertilized eggs develop into females, the organisms are **thelytokous parthenogens**. The unfertilized eggs of **arrhenotokous parthenogens **develop into males, as in the case of the haplodiploid insects of interest in Stouthamer *et al. *[1].

## Some sex or no sex?

An important topic in the evolution of asexuality is the degree to which newly derived parthenogenetic lineages will be reproductively isolated from sexual ancestors. Organisms can have lineages that are parthenogenetic but experience rare sex with males (cyclically parthenogenetic), whereas other lineages may be obligately parthenogenetic. Waterfleas (*Daphnia *spp.), are examples of such organisms. Although some populations consist of cyclic parthenogens, in others meiotic suppressor genes have spread and transformed them to strict parthenogens [6]. In addition, some parthenogenetic vertebrates arising from hybridization show an incomplete loss of sex, and females may still require insemination even if the sperm do not contribute to the genetic composition of offspring.

In the case of symbiont-induced asexuality, an infected female can be in the curious position of being able to reproduce both sexually and asexually. All progeny are daughters, with sexually produced female offspring following from fertilization of haploid eggs and asexually produced daughters arising from unfertilized eggs following chromosome doubling. Obligate asexuality occurs only when infection drives the complete loss of males in the population. This outcome requires two conditions to be met: (i) the bacterium must transmit from mother to daughter with near perfect fidelity, such that all females in the population are infected; and (ii) the diploidization of infected males must occur with high penetrance, such that no males slip through the feminization process. Despite these stringent conditions, the majority of species carrying parthenogenesis-inducing symbionts are obligately asexual and mixed sexual/asexual populations are rare.

This apparent contradiction - that conditions for obligate transition to symbiont-induced asexuality are stringent, but the process occurs commonly - has been addressed in a new model by Stouthamer *et al. *[4]. Their idea is motivated by the observation that a lack of sex in species in which feminizing-symbionts are fixed is associated not just with symbiont presence, but also with loss of sexual function in females [7] (males created through antibiotic exposure are somewhat sexually functional [8]). They [4] provide a pathway for the transition from facultative sex to obligate asexuality that operates through selection. Simply, female receptivity to mating or a female's ability to store and process sperm are degraded by selection to produce sons. This selection acts when the population is still sexual, and is driven by the presence of the female-biased population sex ratio associated with the actions of the symbiont.

Their argument is an elegant one. As a feminizing infection invades, the sex ratio becomes progressively more female-biased. However, sex is still occurring, and natural selection at the level of the individual will act to promote production of sons. In arrhenotokous haplodiploid taxa, male-biased sex ratios can be achieved by females either refusing to mate or not using sperm. An uninfected mother that does not fertilize her eggs will produce only sons, but an infected mother will produce the maximal number of sons possible given the constraints of a feminizing symbiont. A conflict between cytoplasmic genes (favoring *Wolbachia *transmission and daughter production) and nuclear genes (favoring male production by preventing egg fertilization) ensues. Traits favoring production of males will, in an infected mother, in fact be rather ineffective as the symbiont causes the diploidization of unfertilized eggs. Nevertheless, the conflict results in the spread of traits that make sex impossible for females. As a result, the transition to parthenogenesis is irreversible.

## Outcomes of a sexual past and an infected, parthenogenetic future

It is commonly observed that parthenogenetic lineages are short twigs on phylogenetic trees, implying that most are short lived on an evolutionary timescale. This is generally thought to be the result of the inherent low adaptability of asexual populations; such populations have 'difficulty' both in removing deleterious mutations and in recombining advantageous mutations into a single genome. However, it is possible that the low adaptability of asexual populations is particularly acute in recently evolved clones. This may explain the contrast between the few parthenogenetic lineages that are ancient and the majority which are short lived.

Low adaptability of newly parthenogenetic lineages may be driven by the process discussed by Stouthamer *et al. *[4], that is, parthenogenetic lineages contain many useless sexually selected traits. Although the authors [4] argue that directly selected virginity mutations drive the sexual-asexual transition, they note that once a population switches from sexual to parthenogenetic reproduction, many genes for sexual function (for attracting mates, copulation or fertilization) become functionally redundant. If any of these traits are costly, then mutations that erode these functions will be under positive selection, and there will be a series of selective sweeps, each reducing sexual traits that are now costly. Study of *Wolbachia*-infected parthenogenetic populations indeed verifies that morphological and physiological sexual traits shaped previously by male-female coevolution do degrade, but the rate of erosion may vary among traits [9]. Essentially, the older the parthenogenetic lineage or the longer the population has been fixed for infection, the less sexually functional females and males are expected to be.

We cannot currently quantify the relative contributions of positive selection and mutation accumulation to the observed loss of function in the courtship and reproductive systems of recent asexual lineages. It is likely, however, that many redundant sexual traits are costly and the early phase of asexual life is characterized by recurrent bouts of positive selection favoring loss-of-function mutations in the reproductive system. Because asexual populations lack recombination, these selective sweeps will reduce standing genetic diversity. Reduced genetic diversity is thought to put asexual lineages at a great disadvantage when faced with complex or fluctuating environments (such as coevolving parasites [10]) and thus we arrive at a potential explanation for why many parthenogenetic populations are short lived. Overall, positive selection for the removal of redundant sexual traits in newly established asexual lineages is likely to reduce standing genetic variation, which may make the population more susceptible to a range of extinction risks associated with fluctuating biotic and abiotic environments.

Why and how asexuality arises from otherwise sexual species remains, for the most part, an enigma to evolutionary biologists. The pathway modeled by Stouthamer *et al. *[4] potentially resolves the conundrum of obligate asexuality through manipulation by symbionts. Under their model, the combination of symbiont manipulation and natural selection can actually favor obligate asexuality in the short term. However, in the long term, the selective sweeps removing superfluous sexual traits may erode genetic variation and may be critical in increasing the vulnerability of novel asexual lineages to extinction.

## References

[B1] Malik S-B Pightling AW Stefaniak LM Schurko AM Logsdon JM An expanded inventory of conserved meiotic genes provides evidence for sex in *Trichomonas vaginalis* PLoS One 2007 3 e2879 10.1371/journal.pone.0002879 18663385 PMC2488364

[B2] Bell G The Masterpiece of Nature: the Evolution and Genetics of Asexuality 1982 Berkeley, CA: University of California Press

[B3] Simon J-C Delmotte F Rispe C Crease T Phylogenetic relationships between parthenogens and their sexual relatives: the possible routes to parthenogenesis in animals Biol J Linn Soc 2003 79 151 163 10.1046/j.1095-8312.2003.00175.x

[B4] Stouthamer R Russell JE Vavre F Nunney L Parthenogenesis inducing *Wolbachia *ends with irreversible loss of sexual reproduction BMC Evol Biol 2010 10 229 10.1186/1471-2148-10-229 PMC2927591 20667099

[B5] Stouthamer R Luck RF Hamilton WD Antibiotics cause parthenogenetic *Trichogramma *(Hymenoptera/Trichogrammatidae) to revert to sex Proc Natl Acad Sci USA 1990 87 2424 2427 10.1073/pnas.87.7.2424 11607070 PMC53701

[B6] Lynch M Seyfert A Eads B Williams E Localization of the genetic determinants of meiosis suppression in *Daphnia pulex* Genetics 2008 180 317 327 10.1534/genetics.107.084657 18689898 PMC2535684

[B7] Pannebakker BA Schidlo NS Boskamp GJF Dekker L Van Dooren TJM Beukeboom LW Zwann BJ Brakefield PM Van Alphen JJM Sexual functionality of *Leptopilina clavipes *(Hymenoptera: Figitidae) after reversing *Wolbachia*-induced parthenogenesis J Evol Biol 2005 18 1019 1028 10.1111/j.1420-9101.2005.00898.x 16033575

[B8] Russell JE Stouthamer R The genetics and evolution of obligate reproductive parasitism in *Trichogramma pretiosum *infected with parthenogensis-inducing *Wolbachia* Heredity 2010 20442735 10.1038/hdy.2010.48 PMC3183844

[B9] Kraaijeveld K Franco P Reumer BM Van Alphen JJM Effects of parthenogenesis and geographic isolation on female sexual traits in a parasitoid wasp Evolution 2009 63 3085 3096 10.1111/j.1558-5646.2009.00798.x 19659591

[B10] Jokela J Dybdahl MF Lively CM The maintenance of sex, clonal dynamics, and host-parasite coevolution in a mixed population of sexual and asexual snails Am Nat 2009 174 S43 S53 10.1086/599080 19441961

